# Evaluation of Sulfuric Acid-Induced Degradation of Potassium Silicate Activated Metakaolin Geopolymers by Semi-Quantitative SEM-EDX Analysis

**DOI:** 10.3390/ma13204522

**Published:** 2020-10-12

**Authors:** Oliver Vogt, Conrad Ballschmiede, Neven Ukrainczyk, Eddie Koenders

**Affiliations:** Institute of Construction and Building Materials, Technical University of Darmstadt, 64287 Darmstadt, Germany; ballschmiede@wib.tu-darmstadt.de (C.B.); ukrainczyk@wib.tu-darmstadt.de (N.U.); koenders@wib.tu-darmstadt.de (E.K.)

**Keywords:** geopolymer, metakaolin, sulfuric acid attack, SEM-EDX, degradation

## Abstract

Geopolymers are synthesized by mixing powdery solids, rich in amorphous silicon and aluminum species, with an alkaline solution, which leads to the formation of an inorganic alumosilicate network. Their acid resistance is affected by the composition, the porosity, and pore size distribution of the hardened binder as well as the type and concentration of the acidic solution. In the present study, two geopolymer mixtures with varying liquid-to-solid ratios and Si/Al ratios were exposed to a sulfuric acid solution (pH = 1) and analyzed after different durations of exposure (7, 14, 28, 56, and 70 days) by using a light microscope and scanning electron microscopy with energy-dispersive X-ray spectroscopy (SEM-EDX). SEM-EDX elemental mapping was used to evaluate the degradation from depth profiles of silicon (Si), aluminum (Al), and potassium (K) leaching. The results clearly show the leaching kinetics of potassium and the dealumination of the network. The separate consideration of specific reaction steps in the course of degradation, namely the depth of erosion (DE), the depth of deterioration (DD), and the depth of reaction for certain elements (DR(e)), indicate a combination of chemical and diffusion controlled degradation mechanisms.

## 1. Introduction

Geopolymers are synthesized by mixing alumosilicate powders with alkaline solutions. In the process of geopolymerisation, hydroxide (OH^−^ from the alkaline solution hydrolyses soluble silicon (Si) and aluminum (Al) species from the alumosilicate powder [1]. The subsequent polycondensation reaction leads to the hardening of the newly formed alumosilicate network [2], which consists of Si and Al tetrahedrons [3] cross-linked by oxygen bridging bonds [1]. The alkali metals (Na^+^, K^+^) from the alkaline solution are integrated into the network to charge balance the negatively charged aluminum tetrahedrons [4]. Smaller proportions of alkalis that are not integrated into the network remain mobile in the geopolymer pore solution [5]. The alumosilicate network is generally characterized by the Si/Al molar ratio, which according to the Loewenstein-rule [6], has a minimum value of 1.0 [7]. The dissolution of a powdery solid material can also be initialized by using acids like phosphate acids [8,9], which is rarely practiced compared to alkaline activation.

Alkaline activation of slag or other calcium oxide (CaO)-rich raw materials are usually referred to as alkali-activated binders (AAB), which also form alumosilicate networks to a certain degree [10,11]. Nevertheless, hydration products are also part of the final reaction product [12,13] and the reaction itself resembles more the hydration of Portland cement [14]. Therefore, the term geopolymer is generally used to describe CaO-free systems or those with a very low CaO content, which also corresponds to the definition coined by Davidovits [15]. In order to differentiate between geopolymers and AAB’s, classifications were proposed with regard to the type of powdery raw materials [16], as well as with regard to the total calcium content [17]. In the context of this study, the term geopolymer is only used to describe low-CaO systems. If geopolymerisation has to take place at ambient temperature, metakaolin is the most suitable precursor, as it contains a favorable ratio of Si/Al [18,19] and exhibits good reactivity in alkaline media.

The deterioration of concrete based on cement was first mentioned in literature in the year 1990 [20]. In several construction applications like biogas plants [21], sewage plants and sewers [22], and cooling towers of power plants [23,24], the deterioration of concrete is caused by sulfuric acid attack. Due to the sulfuric acid, Portlandit and Calcium silicate hydrate of the hardened binder within the concrete get dissolved and new reaction products like ettringite are built [22], which can further increase the process of deterioration by crack formations and a higher exposed surface of the concrete [25].

The high acid resistance of geopolymers is often mentioned as one of the major advantages of these inorganic binders [5,26]. In many cases, comparative studies with hardened geopolymers and hydrated cement have clearly shown that the degree of degradation of hydrated cement is more pronounced than that of geopolymers [27,28,29,30,31,32], due to the calcium-rich hydration products of cement-based binders [30]. In this context, the differences of mass losses between geopolymers and hardened cement paste after acid exposure can be dramatic [33], and a significantly higher strength loss is found in the case of cement-based binders [34].

Leaching of geopolymer reaction products due to acid attack has been investigated in numerous studies. However, the type and reactivity of powder precursors, variations in liquid-to-solid ratios (l/s), different alkali silicate solutions, and the type and concentration of acidic solution make it difficult to generalize knowledge about the degradation mechanism by comparing results from different publications. The progress of geopolymer corrosion in an acidic environment can vary significantly by applying different powdery precursors [35].

According to Sturm et al. [36], a simplified process of deterioration can be described in three consecutive steps. The leaching of free alkali cations (Na^+^, K^+^) and/or ion exchange of the charge balancing cations integrated into the geopolymer by hydronium (H_3_O^+^) (1st step) is followed by the extraction of aluminum due to the hydrolysis of the Si–O–Al bonds (2nd step) and finally the hydrolysis of Si–O–Si bonds (3rd step).

In literature, studies on acid degradation of geopolymer samples include sulfuric acid [29,31,32,36,37,38,39,40,41,42,43,44,45], nitric acid [37,40], hydrochloric acid [44,46,47,48,49,50], and acetic acid [27,28], usually with a pH in the range of 1 to 3. As powder precursors, fly ash [28,29,31,37,38,39,40,41,42,46] or metakaolin [27,43,44,45,48,49,50] is used. In some cases, further powdery solids are added to the above-mentioned precursors in smaller quantities.

The assessment of geopolymer degradation due to acid attack is carried out by determining the weight loss [28,29,31,38,42], the change of dimensions [40,42], and the residual strength of exposed samples [28,29,31,42]. Further analytical methods comprise Nuclear magnetic resonance spectroscopy (NMR) [51], X-ray diffraction (XRD) [27,28,29,42,43,50], Fourier-transform infrared spectroscopy (FTIR) [29,42,43,50,51], Micro X-ray computer tomography (µXCT) [28], Scanning electron microscopy [29,42,50], and Energy-dispersive X-ray spectroscopy (EDX) [27,31,39,41,51,52].

Evaluating the weight loss after acid exposure to obtain information about the degree of deterioration can be difficult, as the specimen size and geometry will influence the results [40], alongside possible increases in mass due to the formation of new products as a result of precipitation of dissolved species [29].

FTIR has proven to be a useful tool, as it can detect the changes in Si/Al ratios [29,30] of the H_3_O^+^ affected geopolymer network, as well as the extent of newly formed Al-OH groups due to the hydrolysis of Si–O–Al bonds and, therefore, the depolymerization of the geopolymer [47].

In some cases, XRD analysis of acid exposed specimens revealed the formation of Faujasite [43], silica gel [36], and some other crystalline zeolites [29]. EDX measurements on deteriorated samples, in most cases performed as spot analysis, showed changing Si/Al and Na/Al ratios [52], a dealumination of the network [27,30], and a condensation of silicon-rich polymeric ions [30].

The objective of the present study is to use energy-dispersive X-ray spectroscopy (SEM-EDX) elemental mapping of pristine and deteriorated geopolymers exposed to a sulfuric acid solution (pH = 1) over a period of 70 days as a semi-quantitative tool to investigate the rate of degradation of specimens and classify the different layers having different degrees of degradation. Performing SEM-EDX analysis at different times of sulfuric acid exposure (7, 14, 25, 56, and 70 days) and measuring changes in sample geometry, degree of erosion, and deterioration provides useful information about the deterioration process like the depth of erosion, the depth of deterioration, and the depth of reaction for the elements silicon (Si), aluminum (Al), and potassium (K). Replacing conventional concrete for sewers, biogas plants, and cooling towers in power plants by geopolymer concrete could increase the lifetime of these components. To make this possible, the progress of deterioration and the elemental composition after a sulfuric acid attack needs to be fully understood.

## 2. Materials and Methods

### 2.1. Materials

An industrial metakaolin with a density of 2.68 g/cm^3^ a high quartz content (39.6%), and an amorphous amount of 46.0% was used to synthesize the two geopolymer mixtures. Chemical and mineralogical composition, specific surface area, and particle size distribution have been presented in a previous publication [53]. Impurities like quartz reduced the overall reactivity of the metakaolin but could also have a positive effect in the context of the resistance to acid attack, as those particles could partially block the pores and reduce the extent of alkali cations (Na^+^, K^+^) leaching from the geopolymer [50]. In addition, higher amounts of quartz reduced the water demand of the powder, which could significantly influence the total porosity and pore size distribution of the hardened binder. Calculation of the amorphous amounts of Si and Al in metakaolin by considering the chemical and the mineralogical composition of metakaolin, as proposed by Vogt et al. [53], resulted in the amorphous molar Si/Al ratio of the metakaolin to be 0.89.

Metakaolin (NEWCHEM GmbH, Baden bei Wien, Austria) was activated by an industrial potassium silicate solution (Wöllner GmbH, Ludwigshafen, Germany) with a molar SiO_2_/K_2_O ratio of 1.5, pH 13.5, density of 1.51 g/cm^3^, viscosity of 20 mPas, and a solid content of 45%. Even though in some publications it is claimed that potassium silicate solutions reduced the acid resistance of geopolymers [29,40], compared to metakaolin activated with sodium silicate solution, the low viscosity of the alkaline solution used in this study enabled significantly lower l/s ratios with good processability at the same scale.

### 2.2. Geopolymer Samples

Information about the mixing procedure of geopolymer paste can be taken from Vogt et al. [53]. Small prisms (80 mm × 20 mm × 20 mm) were cast in polyethylene molds, compacted on a vibration table until no more air bubbles could be seen on the surface, and cured at ambient temperature (21 °C, 50% RH) for 28 days, before exposing the specimens to sulfuric acid (Carl Roth GmbH + Co. KG, Karlsruhe, Germany) at pH 1. To avoid moisture loss and to keep the samples fully saturated, the molds were wrapped with aluminum adhesive tape, demolded after 1 day, and rewrapped with aluminum tape. To avoid interaction between the aluminum adhesive tape and the sample surface, molds and specimens were wrapped with polyethylene film in a first step before applying the aluminum adhesive tape.

Fully saturated samples guarantee a diffusion-controlled acid attack [28]. The same procedure was performed for larger prisms (160 mm × 40 mm × 40 mm), which were tested for compressive strength after 28 days of ambient curing (21 °C, 50% RH). In addition to the l/s ratios, Table 1 shows the molar Si/Al and K/Al ratios of the two geopolymer mixtures of this study, MK54 and MK60, the number in the designation representing the l/s ratio.

Si/Al_am_ contains Si from potassium silicate solution and amorphous Si and Al from metakaolin. Si/Al_tot_ contains Si from potassium silicate solution and the total amount of Si and Al from metakaolin. K/Al_am_ and K/Al_tot_ contain K from potassium silicate solution and amorphous Al from metakaolin (K/Al_am_) and the total amount of Al from metakaolin (K/Al_tot_), respectively.

Liquid ‘l’ comprises the total mass of potassium silicate solution (water and dissolved alkali-silicates), while ‘s’ represents the total mass of powder metakaolin. Thus the mix designs of MK54 and MK60 contain differing amounts of water and percentages of SiO_2_ and K_2_O from the potassium silicate solution. Expressed in mass per unitary volume, 1 dm^3^ of MK54 paste comprises 1.369 kg of metakaolin and 0.739 kg of potassium silicate solution, respectively 1.298 kg of metakaolin and 0.779 kg of potassium silicate solution for 1 dm^3^ of MK60 paste.

### 2.3. Characterization Methods of Unexposed Samples

To verify the suitability of the 2 geopolymers as potential building materials, setting time and compressive strength were determined. Compressive strength was tested with half prisms (80 mm × 40 mm × 40 mm) at a loading rate of 2.4 kN/s, following the guidelines of DIN EN 196-1 [54]. An automatic Vicat needle instrument (ToniSET One, Toni Technik, Berlin, Germany) was applied for determining initial and final setting time. To quantify the difference in porosity and pore size distribution, mercury intrusion porosimetry (MIP) was conducted with a Pascal 440 Mercury Porosimeter (ThermoFisher, Waltham, MA, USA) on 28 days cured specimens. For MIP measurements, samples were immersed in liquid nitrogen and dried until mass constancy with a freeze drier (Lyotrap, LTE Scientific Ltd., Oldham, UK).

Qualitative powder X-ray diffraction was performed on 28 days cured specimens and after 7, 14, 28, 56, and 70 days of exposure to sulfuric acid, with a Bruker D2 Phaser (Hamburg, Germany). Operating conditions were set to 40 kV and 10 mA, configured with CuKα1,2 radiation and linear LYNXEYE detector, with 5 degrees opening, goniometer measurement circle 283 mm, primary optics with 0.4 mm 220 fixed slit, 2.5 degrees soller slits, and using Ni-filter in secondary optics. All samples were measured from 5 to 70 degrees (2θ) with 0.02 step size and measurement time of 2 s/step. Powder samples of exposed geopolymers for XRD analysis were prepared by crushing and grinding only the deteriorated layer of the exposed samples.

The elemental composition of unexposed samples MK54 and MK60 were investigated by SEM-EDX measurements to calculate the ratios Si/Al and K/Al before exposure to sulfuric acid and to obtain the reference values ref(min) and ref(max), which were used to determine the depth of reaction for a certain element (see Section 2.7). The procedure for sample preparation before SEM-EDX analysis is described in Section 2.5. Section 2.6 contains relevant information about the SEM-EDX measurements.

### 2.4. Exposure to Sulfuric Acid

**Figure 1:**
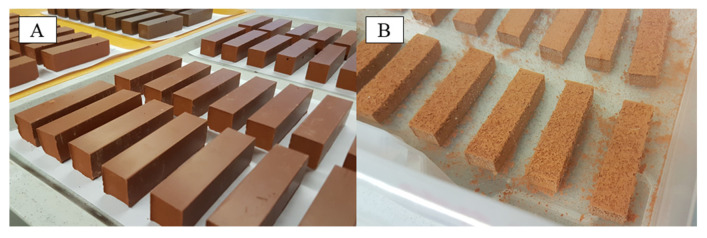
Geopolymer prisms MK54 (80 mm × 20 mm × 20 mm) before (**A**) and after 17 h in sulfuric acid solution (pH 1) (**B**), where the corrosion due to the erosion of the geopolymer specimen surface is already visible.

A Sulfuric acid solution was prepared with deionized water and concentrated sulfuric acid (96%). After curing the geopolymer samples for 28 days, exposure to sulfuric acid solution started at an initial pH of 1 over a period of 70 days. Specimens in the exposure tank had a minimum distance of 1 cm from each other and to the walls of the tank (Figure 1). MK54 and MK60 were stored in separate tanks. The volume ratio of acid solution-to-specimens was 22. During the test, the solution was not exchanged, which corresponds to a real field application, but the pH was kept constant at approximately 1 by adding a 50% concentrated sulfuric acid solution manually at least once a day. The pH of a solution was measured with a pH meter (Hanna pH 211, Vöhringen, Germany) at least once a day, as an average of two replicate setups. Corroded geopolymers were analyzed with different characterization methods after 7, 14, 28, 56, and 70 days of exposure to sulfuric acid. No mechanical stirring of the acid solution was performed, since height-related differences in sulfuric acid concentration were not to be expected, due to the complete dissociation of sulfuric acid, and an acceleration of the degradation process, which should be prevented [55]. Manual stirring of the solution was done daily, before and after the post acidification with 50% concentrated sulfuric acid.

### 2.5. Sample Preparation after Sulfuric Acid Exposure

In order to perform SEM-EDX measurements, specimens were carefully removed after exposure to sulfuric acid and dried at 40 °C until mass constancy. Cross-sections of 1 cm thickness were dry cut with a low-speed diamond-tipped precision cutter (IsoMetTM, Buehler, Esslingen am Neckar, Germany). The cross-sections were impregnated with epoxy resin under vacuum and heated at 40 °C for 24 h to cure the resin. The polishing of specimens was performed with a resin-bonded diamond disc (hardness range HV 150 to 2000) from a polishing machine (LaboForce-100, Struers, Cleveland, OH, USA) to reveal the surface of the specimens (rotational speed 300 rpm). For a second polishing, an automated polycrystalline diamond spray was used at a rotational speed of 150 rpm (9 µm, 3 µm, and 1 µm size).

### 2.6. Characterization of Sulfuric Acid Exposed Samples

The revealed degradation mechanism due to exposure to sulfuric acid was analyzed by measuring the eroded and corroded layer of epoxy embedded specimens. The thickness of the eroded layer was obtained by taking into account the dimensions of specimens before and after exposure to sulfuric acid. The thickness of the deteriorated layer was measured using a light microscope (VHX-600, Keyence, Neu-Isenburg, Germany). Each mean value resulted from eight individual measurements, four values on each side of the specimen (left and right). Elemental mapping, obtained by scanning electron microscopy with energy-dispersive X-ray spectroscopy (SEM-EDX), were used to evaluate the leaching and depolymerization of the alumosilicate network. SEM-EDX investigations were performed with a Zeiss EVO LS25 SEM (Jena, Germany) and an EDX detector (EDAX, Ametek, Berwyn, PA, USA) under low-vac. modus at 0.1 mbar to prevent charging effects on the samples. All samples were studied at 2.0 nA probe size and 15.0 kV accelerating voltage with a 100x magnification for EDX. Elemental mappings were conducted to determine the spatial element distribution of silicon (Si), aluminum (Al), and potassium (K) in the cross-section of sulfuric acid exposed epoxy embedded specimens. Elemental mappings were performed at a dwell time of 200 µs for each pixel (512 × 400 pixels in total) with a repetition rate of 128.

To minimize the influences caused by interactions between the sample surface and electron beam, ZAF correction was applied. The ZAF correction uses an algorithm which accounts for Z—the atomic number, A—the absorption correction, and F—the fluorescence correction. These corrections are used for matrix effects (e.g., differences in mean atomic number, in the absorption of X-rays, and production of X-rays or X-ray fluorescence). In combination with internal standards, an error is quantifiable. However, due to the use of these internal device standards and not sample-specific standards, the results should be considered as semi-quantitative [56].

### 2.7. Position of Elemental Mappings and Terminology of Experimental Results

**Figure 2:**
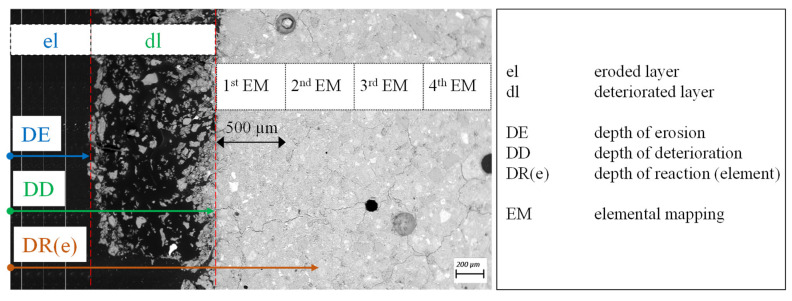
Description of a used terminology: eroded layer (el), deteriorated layer (dl), depth of erosion (DE), depth of deterioration (DD), and depth of reaction for specific elements (DR(e)). The positioning of SEM-EDX elemental mapping (EM) for profile evaluation of the sulfuric acid-induced degradation.

The evaluation and presentation of the experimental results in this study are carried out according to a specific procedure (see Figure 2). The terms “depth of erosion” (DE), “depth of reaction” (DR(e)), and “depth of deterioration” (DD) have already been used by König et al. [28,55], where DD is composed of DE and DR. In the present study, DE, DR, and DD are interpreted differently.

All previously mentioned depths refer to the pristine surface (not exposed to sulfuric acid), i.e., the outer dimensions of the specimen. DE includes the thickness of the eroded layer (el), DD comprises the eroded layer and the deteriorated layer (dl). DR(e) includes DD and the corresponding depth of reaction for a specific element, which will be explained below. DR(e), which results from EDX elemental mapping, was measured for Si (DR(Si)), Al (DR(Al), and K (DR(K)). The first elemental mapping was conducted at the end of DD, where the following mappings approached the core of the specimen. The deteriorated layer was not analyzed by EDX, due to significantly higher porosity and the resulting inaccuracy of the measurements, which was detected by preliminary testing.

**Figure 3:**
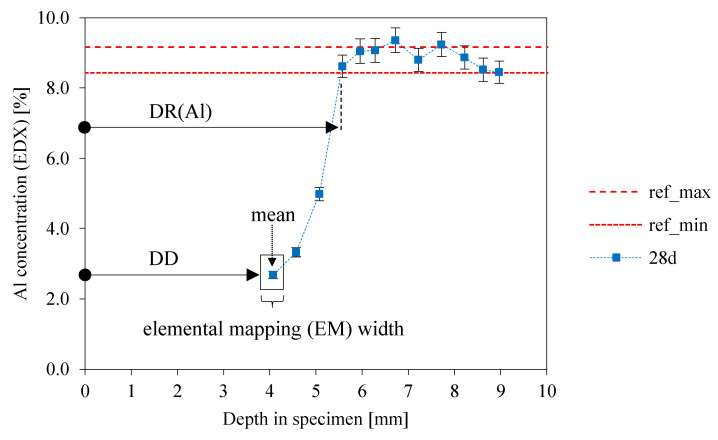
Method of determining the depth of reaction DR(e) of geopolymer elements (SEM-EDX elemental mapping).

Figure 3 illustrates the procedure for determining DR(e), using the example of aluminum. The region of interest along the specimen’s depth was divided into 500 µm sections (“elemental mapping (EM) width”, Figure 3). For each section a mean and corresponding absolute error was calculated, which results in the graphs for DR(e). The red dotted horizontal lines in Figure 3 (ref_max_ and ref_min_) were the minimum and maximum percentages of the element of the original sample (no exposure to sulfuric acid) after 28 days of curing. Minimum and maximum values were obtained by considering the mean value and the corresponding absolute error (i.e., mean ± one standard deviation) from element mapping. DR(e), in this case, DR(Al), was determined by the mean value of the elemental mapping, at which the ref_min_ line in Figure 3 was reached. Using the example of Al, DR(Al) is the spot at a certain specimen depth where the dealumination effect (i.e., the lower percentages of Al in exposed sample area than in the original sample) of the alumosilicate network had ceased, as the Al-concentration reached the values of the unexposed sample.

## 3. Results

### 3.1. Physical Properties of Unexposed Samples

The evolution of compressive strength has been presented in a previous publication [53]. MK54 and MK60 reached 58.2 MPa and 53.6 MPa, respectively, after 28 days. Compressive strength after 1 day revealed that both geopolymer formulations are highly reactive systems, as the values were only slightly smaller compared to 28-day compressive strength (51.4 MPa for MK54, 48.2 MPa for MK60). The initial and final setting for MK54 is 127 min and 147 min, for MK60 160 min and 190 min. Compared to strength and setting, porosity might be more relevant for evaluating the results of sulfuric acid exposed specimens. Table 2 shows the total mercury intruded porosity and pore size distribution of samples after 28 days of ambient curing.

Apart from total porosity, there was a more noticeable difference in pore size distribution. The higher l/s ratio of MK60 results in a shift of the pore size distribution towards bigger pores, which coincided with the results from the literature [57,58]. The biggest change could be observed for pores < 10 µm and in the range of 20 µm to 50 µm, where the shift was most pronounced.

### 3.2. Elemental Mapping of Unexposed Geopolymers

**Figure 4:**
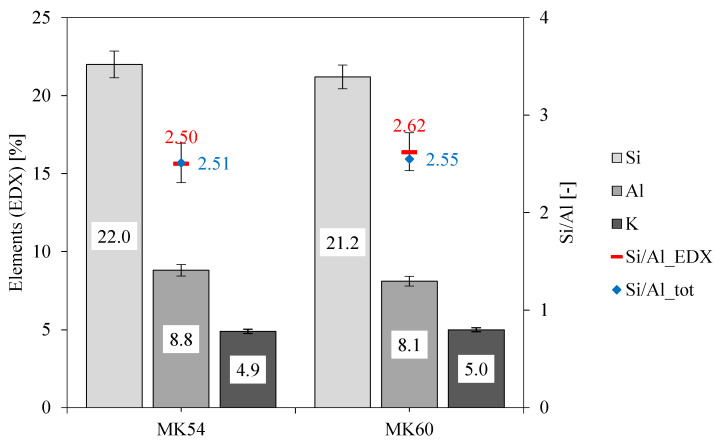
Percentage element distribution for Si, Al, K, and Si/Al ratios obtained from SEM-EDX elemental mapping and from the chemical composition of geopolymer formulation (Si/Al_tot_) for geopolymers MK54 and MK60.

SEM-EDX elemental mapping from unexposed samples was used to evaluate DR(e), for determining the reference values ref_min_ and ref_max_ (Figure 3) and for comparing the SEM-EDX elemental composition with the chemical composition obtained by X-ray fluorescence (Si/Al_tot_, Table 1). Figure 4 reveals the percentage of element distribution for Si, Al, and K, obtained by SEM-EDX.

The relative errors of the elements obtained by SEM-EDX analysis for MK54 were 3.9% (Si), 4.2% (Al), and 2.8% (K), and 3.6% (Si), 3.8% (Al) and 2.5% (K) for MK60. With these values, the absolute errors of elements could be calculated, which were 0.86% (Si), 0.37% (Al), and 0.14% (K) for MK54, and 0.76% (Si), 0.31% (Al) and 0.13% (K) for MK60. Furthermore, mean Si/Al_EDX_ ratios of 2.50 (0.21) for MK54 and 2.62 (0.20) for MK60 could be calculated (absolute errors in brackets), also illustrated in Figure 4 as Si/Al_EDX_.

The higher Si/Al ratio of sample MK60 was due to the lower amount of Al from metakaolin, as the increase of l/s from 0.54 to 0.60 resulted from a lower amount of metakaolin in the formulation. This was also confirmed by the lower amount of Si in MK60 obtained by SEM-EDX measurement.

Since the deviation of Si/Al_EDX_ and Si/Al_tot_ was very small for both geopolymers, ratios obtained by SEM-EDX elemental mapping were considered accurate, especially in the context of ref_min_ and ref_max_ for determining DR(e) (see Figure 3). Calculated K/Al_tot_ ratios from the chemical composition of precursors and mass percentages in the geopolymers led to 0.50 for MK54 and 0.55 for MK60, while ratios obtained by SEM-EDX are 0.56 (0.14) for MK54 and 0.62 (0.13) for MK60 (absolute errors in brackets).

**Figure 5:**
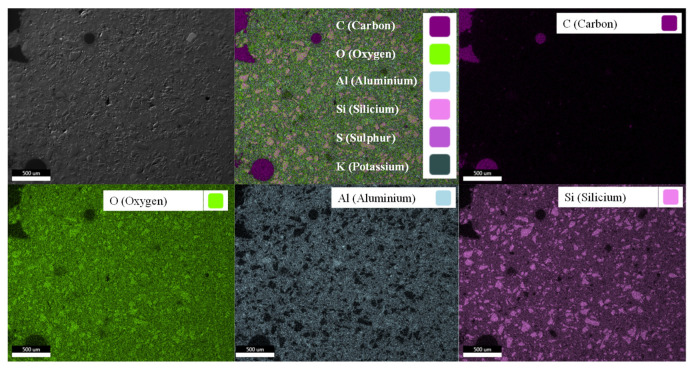
SEM-EDX elemental mapping of epoxy resin embedded cross-section of pristine Geopolymer MK54, i.e., before exposure to sulfuric acid (superposition of elements in the top center image; SEM image in top left image).

The element distribution in MK54 was illustrated in the EDX elemental mapping in Figure 5, showing the superposition of all chosen elements (upper-middle image) as well as elements C (carbon), O (oxygen), Al (aluminum), Si (silicon), S (sulfur), and K (potassium) separately. Superposition of all elements and the C-image showed that carbon is generally associated with large pores or defects in the structure of geopolymer, as those regions are filled with epoxy resin from sample preparation and therefore are detected as carbon by SEM-EDX. Comparing Si- and Al-images indicated that the high quartz content of metakaolin was more or less evenly distributed in the geopolymer gel. Bright purple spots in the Si-image were quartz particles, which were dark spots in the corresponding Al-image, without showing signs of the color representing Al. This proved that the bright purple spots were not a geopolymer reaction product, as this would also be visualized in the Al-image. The quartz particles embedded in the geopolymer gel did not dissolve in an alkaline media, at least not to any relevant extend, and remain mainly as an inert filler within the hardened binder [59,60,61]. This fact led to the large deviations of Si/Al ratios as represented in Table 1. Si/Al_tot_ was calculated from the chemical composition of metakaolin with a quartz content of 39.6%. Si/Al_am_ only considered the amorphous Si and Al from metakaolin. For this reason, the value was close to 1, which corresponded to the Si/Al ratio of pure (meta) kaolinite.

### 3.3. SEM Images of Sulfuric Acid Exposed Samples

**Figure 6:**
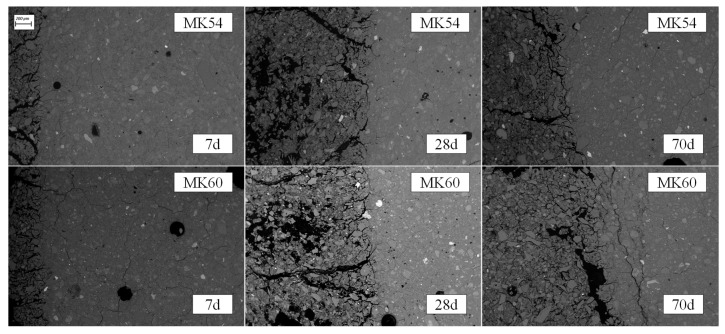
SEM backscatter images of geopolymers MK54 and MK60 after 7 (7 d), 28 (28 d), and 70 (70 d) days of exposure to sulfuric acid, the left side of each image showing the deteriorated layer.

SEM backscatter images of geopolymers MK54 and MK60 after 7, 28, and 70 days of exposure to sulfuric acid are presented in Figure 6. Each of the 6 images clearly showed the deteriorated layer of the geopolymers on the left side of the images, which was characterized by a higher proportion of darker spots due to the higher porosity of these parts of the sample. In addition to the higher porosity, cracks of different sizes were present in each sample’s deteriorated layer. At the depth of deterioration (DD), further cracks were visible, mainly aligned parallel to the transition zone between the deteriorated layer and the apparently intact core of the sample.

Cracks were not only present in the region of the deteriorated layer but also in the apparently intact core of the samples, which may be induced by temperature drying of the specimens before sample preparation with epoxy resin and EDX measurements. Due to the high viscosity of metakaolin geopolymer pastes, the complete removal of air entrapments in the fresh paste by applying a vibration table could not be achieved, which can be seen at the occasional dark spots in the region of the apparently intact core, representing air pores.

### 3.4. Elemental Mapping of Sulfuric Acid Exposed Geopolymers

**Figure 7:**
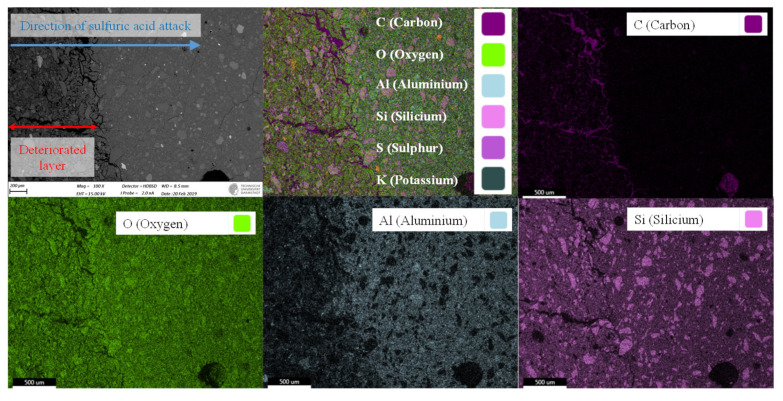
SEM-EDX elemental mapping of epoxy resin embedded cross-section of Geopolymer MK54 after exposure to sulfuric acid, showing high porosity within the deteriorated layer as well as varying element composition due to sulfuric acid attack.

Elemental mapping of sulfuric acid exposed geopolymer samples (Figure 7) showed deterioration of the geopolymer surface exposed to sulfuric acid (upper left image), with higher porosity and micro-cracks.

Superposition of all elements (upper-middle image) and C-image visualized the acid attack induced porosity, as these areas were filled with epoxy resin from the sample preparation. Superposition of all elements and Si-image also suggested that quartz particles were not dissolved significantly due to sulfuric acid exposure. Comparing the Al-image with the Si-image gave a first impression of the deterioration process. Within the deteriorated layer, Al was present to a lesser extent, visualized by differences in color between the deteriorated layer and the core of the sample. Those differences in color were not visible in the Si-image, indicating that Si was far less dissolved then Al.

### 3.5. pH of Sulfuric Acid Solution

**Figure 8:**
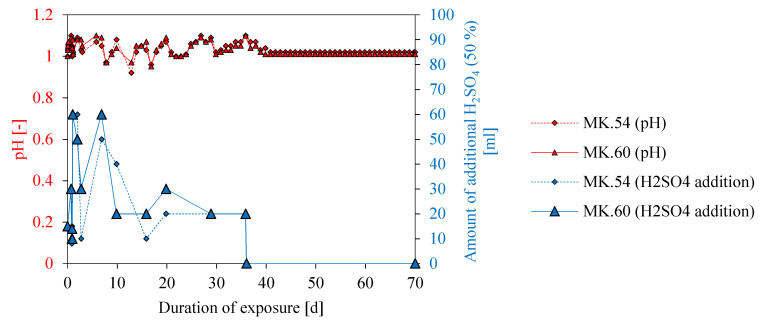
pH of the sulfuric acid solution for MK54 and MK60 over the complete exposure time (70 days) and amount of sulfuric acid (50%) addition to maintain a constant pH in the range of 1.

The initial pH 1 of the sulfuric acid solution could not be maintained constant for the entire duration of specimen exposure as certain fluctuations could not be avoided (Figure 8). Within the first 36 days of exposure, the pH of the solution increased daily, which was compensated by the addition of 50% sulphuric acid. As a result, the pH value could be kept almost constant in a range of 0.9–1.1. Apart from a few single days, pH was always above 1.0. The mean pH over 70 days of exposure was 1.03 for both geopolymer formulations. The steady increase of pH after post-acidification illustrated the neutralization of sulphuric acid by the alkaline pore solution of the geopolymer.

Due to the interaction of the alkaline pore solution and acidic solution, H^+^ of the acidic solution got consumed. As sulfuric acid is a strong acid without buffering capacity, in this case, the acidic solution got neutralized and must be post acidified to keep the pH at a constant level. [55] At the beginning of the sample exposure in sulfuric acid, large amounts of 50% sulfuric acid had to be added (see Figure 8), indicating a strong neutralization reaction. Lower amounts of 50% sulfuric acid for post acidification at later stages of exposure resulted from the extraction of samples for SEM-EDX analysis (7, 14. 28, 56, and 70 days), as well as the lower neutralization rate of the samples due to the initially higher leaching of potassium [45]. Leaching of Si and Al from the geopolymer samples could also neutralize the pH of sulfuric acid, as Si and Al could react with OH^-^ to form Si(OH)^4^ Al(OH)^3^ and other speciation complexes in solution. However, the consumption of OH^-^, in this case, might be low. [62] Post-acidification was stopped after 36 days, as the pH remained constant until the end of exposure (70 days).

### 3.6. Eroded and Deteriorated Layer

**Figure 9:**
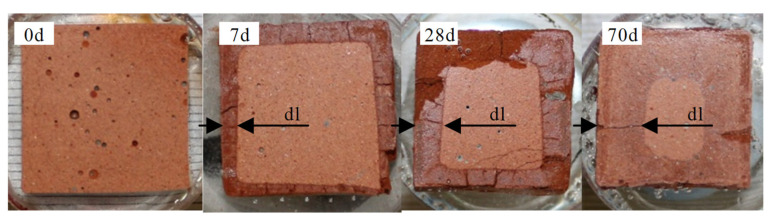
Epoxy embedded samples of geopolymers MK54 for measuring the eroded layer (el) and the deteriorated layer (dl), before (0 d), and after sulfuric acid exposure (7 d, 28 d, and 70 d).

Figure 9 illustrates the epoxy embedded and unexposed sample (0 d) of MK54, and the sulfuric acid exposed samples (7, 28, and 70 days) with the clearly visible deteriorated layer (dl). The deteriorated layers were characterized by a higher porosity, visual cracks, and a different color intensity compared to the core of the samples. Cracks in the deteriorated layer have also been reported in other publications and were explained by shrinkage of the deteriorated layer [40,47]. Whether the shrinkage occurred during exposure to sulfuric acid or subsequent drying at 40 °C during sample preparation, could not be stated clearly in the present study.

**Figure 10:**
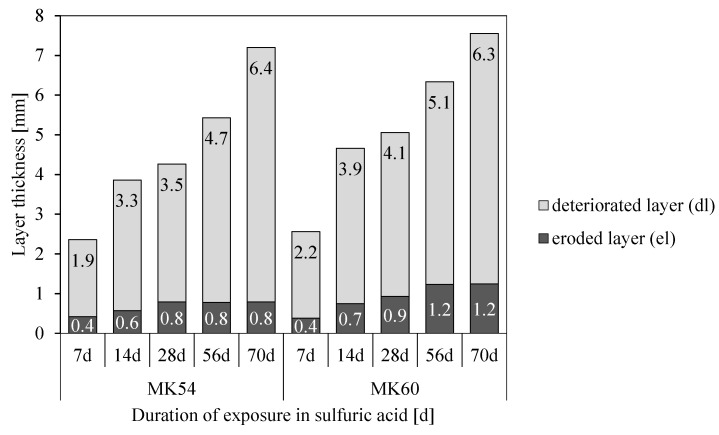
Thickness of eroded (el) and deteriorated (dl) layer of MK54 and MK60 after different exposure periods in sulfuric acid.

The progress of the eroded (el) and deteriorated (dl) layer over the entire exposure period is presented in Figure 10. For MK54, relevant changes in the eroded layer happened within the first 28 days of acid exposure. After 28 days, the eroded layer remained constant, meaning that the dimension of the geopolymer sample did not change further.

MK60 showed a similar trend, whereby a constant value (1.2 mm) of the eroded layer was reached after 56 days. For both geopolymers, the deteriorated layer increased continuously over the whole exposure period, with only minor differences between MK54 and MK60. The progress of deterioration could be subdivided into three stages. A steep rise in values up to 14 days of exposure (1st stage), a transition stage where the deterioration changes only slightly (2nd stage), and a moderate increase after 28 days (3rd stage).

### 3.7. Powder X-ray Diffraction of Unexposed Specimen and Deteriorated Layer

**Figure 11:**
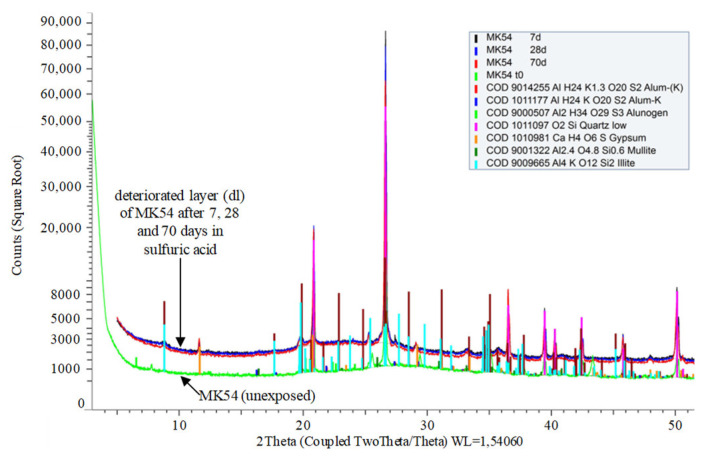
Characteristic parts of XRD patterns of unexposed geopolymer MK54 and deteriorated layers (dl) of MK54 after 7, 28, and 70 days of exposure to sulfuric acid.

Comparing powder X-ray diffraction patterns of unexposed geopolymers and the deteriorated layers of MK54 and MK60 reveal new crystalline phases (gypsum, alunogen, alum-(K)). These phases occur due to the exposure of specimens to sulfuric acid at pH 1, as presented in Figure 11 for MK54 and the deteriorated layers after 7, 28, and 70 days of sulfuric acid exposure.

Traces of mullite, illite, and quartz resulted from the mineralogical composition of the metakaolin. The newly built crystalline phases, gypsum, alunogen, and alum-(K), were detected in all deteriorated layers (7, 14, 28, 56, and 70 days of acid exposure) of MK54 and MK60. Compared to the unexposed samples, XRD patterns of the deteriorated layers indicated a shift of the amorphous hump towards lower angle 2θ, not showing a notable difference between different durations of sulfuric acid exposure for MK54 and MK60.

### 3.8. SEM-EDX: Depth of Reaction DR(e)

**Figure 12:**
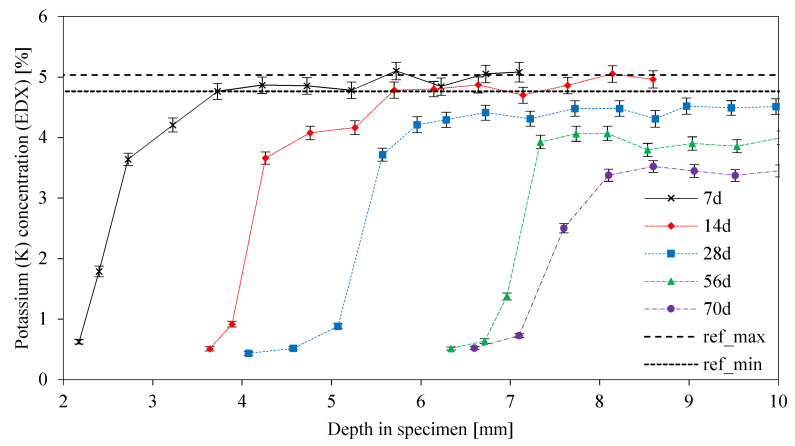
MK54 potassium concentration from EDX elemental mapping, as a function of depth in the specimen. Curves for different exposure times up to 70 days and reference lines ref_min_ and ref_max_ from the original unexposed geopolymer sample.

Figure 12 shows the results from EDX elemental mapping for potassium (MK54). The abscissa of the graph, representing the depth into the specimen, ran from *x* = 0 mm (original surface of the sample) to *x* = 10 mm (i.e., the core of the 20 mm × 20 mm cross-section sample). Each curve (representing different exposure times in sulfuric acid) started at concentrations of about 0.5%. These values were significantly lower than the percentages of the original unexposed sample, in Figure 12 represented by ref_min_ (4.8%) and ref_max_ (5.0%), demonstrating the leaching of potassium out of the geopolymer. The shift of the curves to the right represented the progress of the deteriorated layer, as described in Figure 2 and Figure 3.

After 7 and 14 days of acid exposure, the ref_min_ concentration of the unexposed sample was reached again after a distance of approximately 2 mm from the end of DD. As a result of this, the leaching of potassium had not reached the core of the specimen. The maximum potassium concentration of sample 28 d (4.5%) did not reach ref_min_, indicating a partial leaching of potassium up to the core of the sample. The maximum potassium concentrations of the samples 56 d (4.1%) and 70 d (3.7%) showed that the leaching of potassium continued. Maximum potassium concentrations for 28 d, 56d, and 70 d samples of MK60 were 5.1%, 4.6%, and 3.7%.

**Figure 13:**
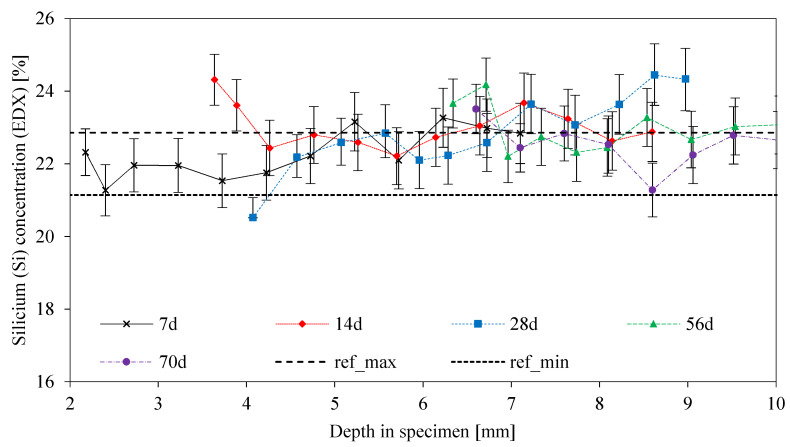
Silicon concentration from EDX elemental mapping, as a function of depth in the specimen. Curves for different exposure times up to 70 days and reference lines ref_min_ and ref_max_ from the original unexposed geopolymer sample.

The Si concentration of MK54 is illustrated in Figure 13. Based on the measured Si concentrations, no leaching of this element could be detected, a fact that has already been discussed before, in the context of Figure 7. It was noticeable that all curves run above the ref_min_ line, therefore representing percentages in the range of the original unexposed geopolymer. Furthermore, the percentages of Si were, in many cases, slightly higher than the percentage of ref_max_, meaning that the Si concentration in some areas might be higher than the concentrations in the original values. As SEM-EDX measurements represented semi-quantitative values, the slightly higher Si concentrations of MK54 should not be interpreted as an accumulation of silicon-rich species, as mentioned by Sturm et al. [36].

**Figure 14:**
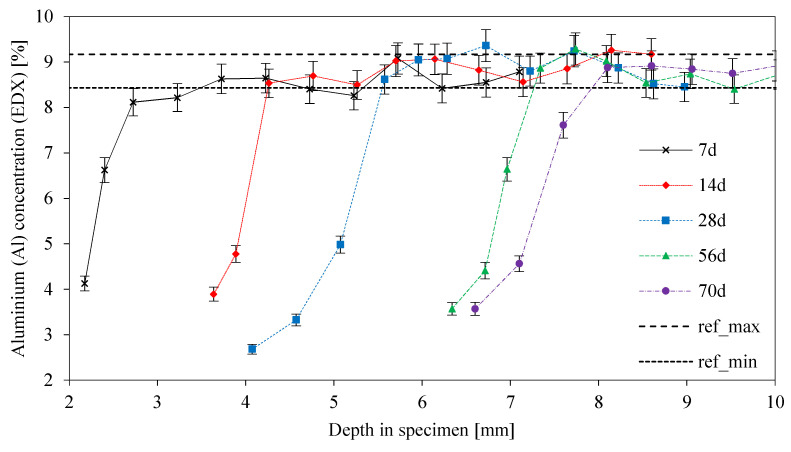
Aluminum concentration from EDX elemental mapping, as a function of depth in the specimen. Curves for different exposure times up to 70 days and reference lines ref_min_ and ref_max_ from the original unexposed geopolymer sample.

Al concentrations as a function of the depth at the specimen (Figure 14) clearly showed the dealumination of a geopolymer as a result of the sulfuric acid attack. Starting at a concentration of approximately 4% (apart from sample 28d), the percentage range of the original unexposed sample with a minimum percentage of 8.43% (ref_min_) and a maximum percentage of 9.17% (ref_max_) was reached after 1–2 mm, depending on the duration of sulfuric acid exposure. As the ref_min_ percentage was reached in all cases (7, 14, 28, 56, and 70 days), the dealumination of the geopolymer had not reached the core of the specimens. The appearance of the curves was also very similar to the course of the K-concentration curves, which suggested a connection between K and Al leaching during the degradation process.

**Figure 15:**
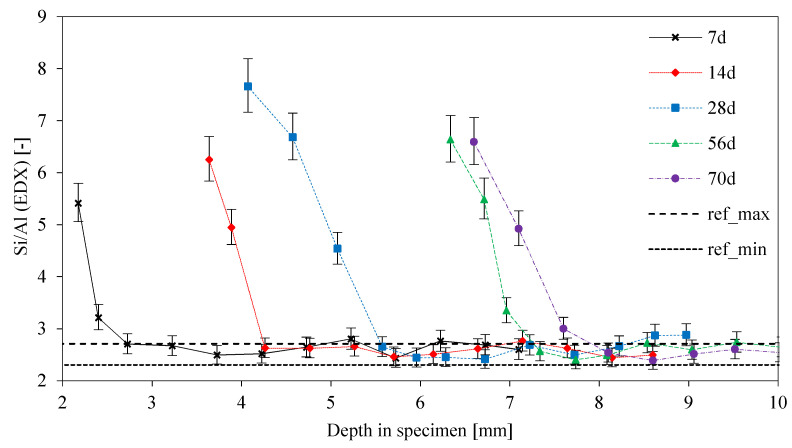
Si/Al ratios calculated by aluminum and silicon concentration from EDX elemental mapping, as a function of depth in the specimen. Curves for different exposure times up to 70 days and reference lines ref_min_ and ref_max_ from the original unexposed geopolymer sample.

In order to better assess the semi-quantitative EDX results, Si/Al ratios were calculated from the individual Al and Si elemental mapping results (Figure 15). The degradation of the geopolymer network led to changes in the Si/Al ratio, as the Al percentages decrease due to the sulfuric acid attack and the Si percentages only slightly changed. Ratios in the interfacial transition zone between the deteriorated layer and visually intact core were in the range of 6–8, reaching the range of the original sample (ref_min_, ref_max_) within a distance of less than 1 mm. Higher Si/Al ratios were also reported by Bakharev [29] and Baščarević et al. [52].

## 4. Discussion

**Figure 16:**
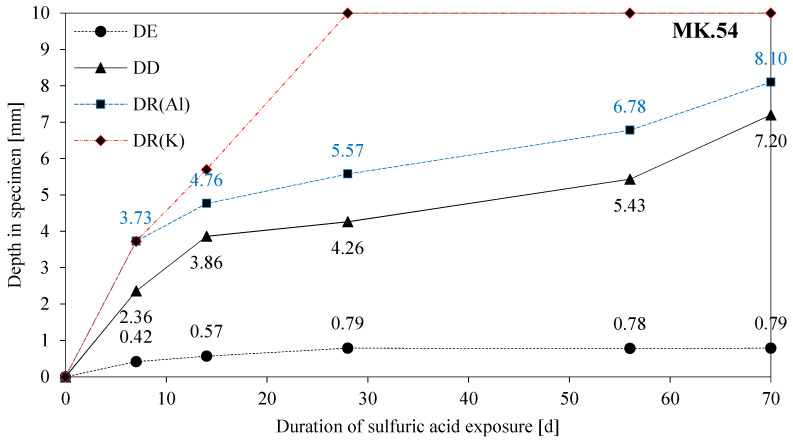
DE (depth of erosion), DD (depth of deterioration), and depth of reaction of aluminum (DR(Al)) and potassium (DR(K)) for MK54, as a function of the duration of exposure in sulfuric acid.

**Figure 17:**
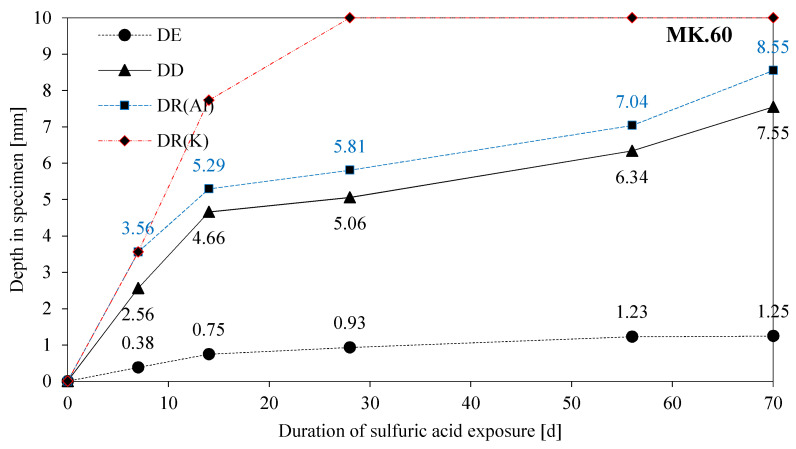
DE (depth of erosion), DD (depth of deterioration), and depth of reaction of aluminum (DR(Al)) and potassium (DR(K)) for MK60, as a function of the duration of exposure in sulfuric acid.

The degradation of geopolymers in this study is investigated under the observed mechanisms of erosion (DE), deterioration (DD), and reaction (DR(e)), and the discussion of results, in particular, takes into account possible interactions between the three mechanisms. To enable this, DE, DD, DR(Al), and DR(K) are presented together in Figure 16 for MK54 and Figure 17 for MK60, where the latter has not been discussed yet, as all of the SEM-EDX results focused on MK54 so far. For MK54 and MK60, the thickness of the eroded layer increases to a certain value and stays more or less constant after 28 days of exposure (MK54) and 56 days of exposure (MK60), respectively. A reason for this could be the formation of the deteriorated layer.

Lloyd et al. [40] tried to explain this by a two-stage degradation process, which is characterized by chemical reactions at the contact zone between sample and acid solution (1st stage) and a diffusion-controlled process (2nd stage), where the latter is resulting from the formation of a reaction layer which is degraded but not completely dissolved. This reaction layer, chemically stable in the range pH 1–3, seems to act as a barrier but nevertheless cannot prevent further deterioration [40].

In the present study, the thickness of the deteriorated layer (dl) increases over the whole period of sulfuric acid exposure, meaning that physical (porosity and cracks) and chemical (leaching and dealumination) changes are an ongoing kinetically driven process with time. However, the gradient of the DD curve up to 14 days of acid attack is much steeper than the gradient after 14 days. The same effect can be observed at the DE curve. This is in agreement with the explanation given by Lloyd et al. [40], as the reaction layer, which is either the whole deteriorated layer (dl) itself or somewhere inside dl, slows down the deterioration as well as the erosion process. The fact that DE does not change after 28 days (MK54) and 56 days (MK60), respectively, and that DD gradually proceeds might be due to the stability of the reaction layer in the pH range of 1–3. The increase of the DD curves after 56 days of exposure might be induced by the formation of cracks in the deteriorated layer, as the cracks will affect the diffusion barrier of the deteriorated layer and progresses the sulfuric acid faster and to a higher extent [63].

Comparing DE and DD of MK54 and MK60 shows that the erosion and deterioration process is progressing much faster for the geopolymer with the higher l/s ratio (MK60). It can be stated, that in the present study the initial water content of the geopolymer, which influences porosity and pore size distribution of the hardened binder, is the decisive factor for the acid resistance of geopolymers. The higher l/s ratio of MK60 results from the higher amounts of potassium silicate solution, which implicitly leads to a slight increase of the Si/Al ratio of the mixture (see Table 1). Due to the higher water amount of geopolymer MK60, a higher volume of pores remain in the hardened binder. Higher amounts of Si increases the resistance against acid attack [40,64], probably due to a denser network and a higher volume of geopolymer gel. Nevertheless, the better stability of geopolymers with higher Si/Al ratios in acidic solutions also results from the deterioration process itself, namely the dealumination.

A network with Si/Al = 1 contains only Q^4^(4Al) species, meaning that each Si atom is connected to four Al atoms by oxygen bridging bonds. In the case of dealumination (hydrolysis of Si-O-Al bonds), this leads to the complete destruction of the alumosilicate network [36]. At higher Si/Al ratios, more Si atoms are connected via bridging oxygens, which gives a residual stability of the matrix after dealumination, respectively less mass loss and higher residual strength [32].

As MK60 performed worse than MK54 in sulfuric acid, even with a higher Si/Al ratio, the porosity and pore size distribution must be the factor determining the degradation rate. The difference in total Hg intruded porosity (see Table 2) is only marginal, whereas the shift to bigger pores due to higher l/s might be the decisive factor, a fact that Bakharev [29] and Lloyd et al. [40] observed as well.

The cracks in all of the samples’ deteriorated layers and those present in the initial regions of the apparently intact core may also have an influence on the progress of corrosion. However, cracks were only detected after drying the specimens for subsequent sample preparation and EDX elemental mapping. As a consequence, no statements can be made about the effects of possible cracks.

Comparing DR(Al) and DR(K) in Figure 16 gives reason to believe what Lloyd et al. [40] described as a two-staged degradation process. The chemical reaction, in the present study the dealumination of the geopolymer, dominates the degradation process within the first seven days of exposure, as DR(Al) and DR(K) are almost identical after seven days of exposure. After 14 days, DR(K) has progressed deeper into the geopolymer sample than DR(Al), an effect that is even more pronounced for MK60. This indicates that the deterioration mechanisms after 14 days of exposure are diffusion-controlled, as the reduction of K concentration due to sulfuric acid intrusion progresses, although the dissolution of Al stops in shallower layers of the sample.

This fact is also discussed by considering the dissolution process. Leaching experiments with geopolymer powder, in many cases, reveal that the alkalis are most easily dissolved [51,62]. Regarding the alkalis, the leaching of K^+^ from the geopolymer includes the exchange (or neutralization) of pore solution and the leaching from the charge balancing alkalis of the alumosilicate network [62]. As the neutralization probably will not affect the alumosilicate network of the geopolymer, so long as the drop in pH is not too severe, the concentration of K decreases further without a dealumination of the network. Even if the drop in K concentration results from leaching of K^+^ from the charge balancing cations of the alumosilicate network, the possible cation exchange within the network could prevent the dealumination of the network. The cation exchange can be done by hydronium ions (H_3_O^+^), which maintain the charge balance [47,49,64].

The very similar curves of DD and DR(Al) prove, that the destruction of the geopolymer is caused by the dealumination of the alumosilicate network [27,29] since the progress of DR(Al) precedes DD by approximately the same distance over the whole period of acid exposure.

The curve of DD and DR(Al) with the large gradient within the first 14 days of exposure and the following drop of the gradient suggests, that some pore-blocking effects by newly formed species must have occurred. Formations of zeolites after the acid attack have been reported in previous publications [29,43], not mentioning a positive effect but rather a deterioration which led to a loss in mechanical strength. Furthermore, the calcium-containing zeolite Na-P1 (gismondine), which was detected by Bakharev [29], originated in the CaO content of the fly ash (CaO content 3.5%), the zeolites from the faujasite family detected by Palomo et al. [43] resulted from the exposure of metakaolin geopolymer in seawater and acetic acid.

Due to the dealumination and depolymerization of the network (breaking of Si–O–Al and Si–O–Si bonds), Si–OH and Al–OH groups in solution emerge [29]. At a very low pH of the acidic solution, a new condensation of polymeric ions rich in silica can follow the depolymerization of the alumosilicate network—the extent of the reaction depending on the source of alkaline activator and the temperature [29,30]. A pore-blocking effect and thus obstruction of a further acid attack, induced by precipitated silica, is mentioned by Sturm et al. [36], who also claim that the pore-blocking effect can only occur if the pores are small enough. Silicic acid as a new phase formed by the sulfuric acid exposure of the geopolymer was detected by Sturm et al. [36], Fernández-Jiménez et al. [30], and Bakharev [29]. Silicic acid ((SiO_x_(OH)_4−2x_)_n_) is most stable in the range of pH 2–3 [65]. However, in the case of a supersaturated solution, amorphous silica can be formed as either colloidal particles, as precipitation, or as gel [29]. The change in gradients of the DD and DR(Al) curves in Figure 16 and Figure 17 could be explained by such silica-rich, pore-blocking new phases. Under the assumption of an early pore-blocking effect, the deterioration would be slowed down, which could be the reason for the gradient change after 14 days of exposure. The newly precipitated silica phases arise from the breaking of Si–O–Al and Si–O–Si bonds. Furthermore, the SEM-EDX elemental mappings clearly revealed a decrease in Al- but not in Si-concentration. It can be concluded that the dissolved Al species diffuse towards the surface of the deteriorated layer and enter the sulfuric acid solution, whereas the dissolved Si species remain inside of the deteriorated layer.

Nevertheless, certain amounts of the dissolved Al will also form new phases and remain in the deteriorated layer, as the newly built aluminum sulfate mineral alunogen (Al_2_(SO_4_)_3_^.^17H_2_O) and the potassium aluminum sulfate mineral alum-(K) (KAl(SO_4_)_2_^.^12H_2_O) in the XRD patterns of the deteriorated layers reveal. This could also lead to a pore-blocking effect. The low intensity of the gypsum peak in the XRD patterns of the deteriorated layers suggests, that even with a very low CaO content of the metakaolin, which is 0.8% [53], gypsum can be built. Whether this low amount has an influence on the pore-blocking effect or cracking of the deteriorated layer cannot be said with certainty. The shift of the amorphous hump in the XRD pattern, which is characteristic for the amorphous geopolymer gel, can be interpreted as the partially dissolved geopolymer gel, which has been previously reported by Zhang et al. [42].

Potassium concentrations from SEM-EDX elemental mapping of unexposed sample and sulfuric acid exposed samples are presented in Table 3. The minimum and maximum value for the unexposed sample originate from the mean percentage and corresponding absolute error. The minimum value of each acid exposed sample is the mean of the first elemental mapping taken after DD (see Figure 2). The maximum value of each acid exposed sample represent the highest mean value from elemental mappings taken further inside the core of exposed samples.

As already mentioned and shown in Figure 16 and Figure 17, the potassium concentrations of the 7 d and 14 d samples reach the ref_min_ of the unexposed sample. For the samples 28 d, 56 d, and 70 d, the leaching of potassium reached the core of the sample which resulted in percentages lower than ref_min_. The 7 d and 14 d samples show approximate maximum values, the progress of leaching up to the core for longer durations of sulfuric acid exposure shows higher potassium percentages for MK60. MK54, with a lower amount of potassium silicate solution, contains fewer alkalis than MK60. The neutralization of the alkalinity in the geopolymer is therefore accelerated due to lower amounts of alkalis [37], which should reduce the degradation depth according to Lloyd et al. [40]. Under the assumption that the neutralization is the first step in the degradation process of geopolymers, MK60 should be more resistant against acid attack because more alkalis are present to neutralize the sulfuric acid solution. Nevertheless, comparing DD and DR(Al) in Figure 16 and Figure 17 suggests the contrary, as for each exposure duration, DD and DR(Al) values of MK60 are higher than those of MK54.

Considering the two-stage degradation process from Lloyd et al. [40] and assuming that after 14 days of exposure a diffusion-controlled degradation mechanism dominates, the further increase of DD and DR(Al) are analyzed next. DD and DR(Al) of the 14d sample and the percentage increase of DD and DR(Al) starting from 14d samples are listed in Table 4.

DD and DR(Al) of MK60 14 d sample progressed into deeper zones than in the case of MK54, however, the following percentage increase of DD and DR(Al) is less pronounced in the case of MK60. This may lead to the conclusion, that the chemical reaction (1st stage of degradation process) up to 14 days of exposure is most influenced by the higher porosity and the bigger pores of the geopolymer MK60, meaning there is less solid material in the bulk volume of the geopolymer that needs to be dissolved. The diffusion-controlled reaction (2nd stage of degradation process) after 14 days of exposure may be most influenced by the total amount of free alkalis in the pores of the geopolymer—a higher neutralization potential—which will slow down further degradation processes.

Although the above-mentioned statements on the two-stage degradation process are only hypotheses, they are in agreement with the clearly observable influence of porosity and pore size distribution. The porosity of (low CaO) geopolymers is characterized by a high degree of connectivity [57] and depends mainly on the amount of water in the mixtures [57,66], the Si/Al ratio of the network, and the type of alkaline activator solution [67,68]. However, due to the ink-bottle effect, mercury intruded pore size distribution does not represent the pore size but the pore entry size [69]. Therefore, larger pores are often not detected by MIP measurements, especially in the case of geopolymers [67]. This makes the correlation between the MIP pore size distribution and the degree and progress of degradation a difficult task.

## 5. Conclusions

Scanning electron microscopy with energy-dispersive X-ray spectroscopy (SEM-EDX) is a useful tool to semi-quantify the progress of sulfuric acid-induced degradation in geopolymer materials, especially when the element concentration curve within the corroded sample needs to be analyzed. The results of the two geopolymer mixtures with slightly different l/s and Si/Al ratios show significant differences in the degree of degradation. The results allow the following conclusions:

1. The depth of erosion (DE) of the acid exposed surface of specimens increases up to 28 days (MK54) and 56 days (MK60) without any relevant further increase in values after the aforementioned durations;
2. The gradient of the DD-curve (depth of deterioration) is steep up to 14 days of exposure with a lower gradient after that point. The same effect can be observed for the depth of reaction for aluminum (DR(Al));
3. The curve for the depth of reaction of potassium (DR(K)) is similar to the DR(Al) curve up to 7 days of exposure, with steeply rising values after 7 days;
4. The gradients of DE, DD, and DR(Al) indicate a two-stage degradation mechanism (initially chemically followed by a diffusion-controlled), as already proposed by Lloyd et al. [40];
5. The DR(K) and DR(Al) values indicate a leaching of potassium in the first step of degradation, followed by the dealumination and depolymerization of the geopolymer network;
6. Stronger deterioration of the MK60 sample within the first 14 days of exposure might be induced by the higher porosity and coarser pore size distribution of MK60;
7. After 14 days of exposure, the lower degradation rate (DR(Al) and DD) of MK60 compared to MK54 might result from the diffusive controlled degradation mechanism after that time point and the higher amount of alkalis in the MK60 sample;
8. After sulfuric acid exposure, newly built (potassium) aluminum sulfate minerals in the deteriorated layer like alunogen and alum-(K) indicate a possible pore blocking effect.

## Figures and Tables

**Table 1:** Liquid/solid ratios (l/s), molar Si/Al ratios (Si/Al_tot_: total amount of Si and Al from metakaolin; Si/Al_am_: amorphous Si and Al of metakaolin) and molar K/Al ratios (K/Al_tot_: total amount of Al from metakaolin; K/Al_am_: amorphous Al of metakaolin) of MK54 and MK60. Chemical composition and amorphous amount of Si and Al for Si/Al ratios were taken from Vogt et al. [53].

Name	l/s [-]	Si/Al_am_ [-]	Si/Al_tot_ [-]	K/Al_am_ [-]	K/Al_tot_ [-]
MK54	0.54	1.37	2.51	0.64	0.50
MK60	0.60	1.42	2.55	0.71	0.55

**Table 2:** Total Hg intruded porosity and percentage of pore sizes (MIP) for MK54 and MK60 after 28 days of ambient curing (21 °C, 50% RH).

Name	Total Porosity	Pore Size Distribution by Volume Ratio [%]			
	[%]	<10 nm	10 nm–20 nm	20 nm–50 nm	50 nm–100 nm
MK54	25.6	31.4	36.8	13.1	15.9
MK60	26.4	28.3	30.6	27.3	13.6

**Table 3:** Minimum and maximum potassium percentage of unexposed and sulfuric acid exposed samples, from SEM-EDX elemental mapping.

Name	Potassium Percentage from SEM-EDX Elemental Mapping [%]											
	Unexposed		7 d		14 d		28 d		56 d		70 d	
	ref_min_	ref_max_	Min	Max	Min	Max	Min	Max	Min	Max	Min	Max
MK54	4.8	5.0	0.6	5.1	0.5	5.1	0.4	4.5	0.5	4.1	0.5	3.7
MK60	4.9	5.1	0.5	5.1	0.6	5.0	0.6	5.1	0.6	4.6	0.5	3.7

**Table 4:** DD and DR(Al) of 14 d sulfuric acid exposed geopolymer samples and the percentage increase of DD and DR(Al) starting from the 14 d exposed sample for exposed samples 28 d, 56 d, and 70 d.

Name	14 d Sample		Percentage Increase, Starting from 14 d Percentage [%]					
	[mm]		DD			DR(Al)		
	DD	DR(Al)	28 d	56 d	70 d	28 d	56 d	70 d
MK54	3.9	4.8	10.4	40.7	86.5	17.0	42.4	70.2
MK60	4.7	5.3	8.6	36.1	62.0	9.8	33.1	61.6
